# Construction of a Multiplex Promoter Reporter Platform to Monitor *Staphylococcus aureus* Virulence Gene Expression and the Identification of Usnic Acid as a Potent Suppressor of *psm* Gene Expression

**DOI:** 10.3389/fmicb.2016.01344

**Published:** 2016-08-30

**Authors:** Peng Gao, Yanli Wang, Iván Villanueva, Pak Leung Ho, Julian Davies, Richard Yi Tsun Kao

**Affiliations:** ^1^Department of Microbiology, Li Ka Shing Faculty of Medicine, The University of Hong KongHong Kong, Hong Kong; ^2^Li Ka Shing Faculty of Medicine, The Research Centre of Infection and Immunology, The University of Hong KongHong Kong, Hong Kong; ^3^Department of Microbiology and Immunology, The University of British ColumbiaVancouver, BC, Canada; ^4^State Key Laboratory for Emerging Infectious Disease, The University of Hong KongHong Kong, Hong Kong

**Keywords:** MRSA, anti-virulence, virulence factors, beta-lactams, bacterial infection

## Abstract

As antibiotic resistance becomes phenomenal, alternative therapeutic strategies for bacterial infections such as anti-virulence treatments have been advocated. We have constructed a total of 20 *gfp*-*luxABCDE* dual-reporter plasmids with selected promoters from *S. aureus* virulence-associated genes. The plasmids were introduced into various *S. aureus* strains to establish a *gfp-lux* based multiplex promoter reporter platform for monitoring *S. aureus* virulence gene expressions in real time to identify factors or compounds that may perturb virulence of *S. aureus*. The gene expression profiles monitored by luminescence correlated well with qRT-PCR results and extrinsic factors including carbon dioxide and some antibiotics were shown to suppress or induce the expression of virulence factors in this platform. Using this platform, sub-inhibitory ampicillin was shown to be a potent inducer for the expression of many virulence factors in *S. aureus*. Bacterial adherence and invasion assays using mammalian cells were employed to measure *S. aureus* virulence induced by ampicillin. The platform was used for screening of natural extracts that perturb the virulence of *S. aureus* and usnic acid was identified to be a potent repressor for the expression of *psm*.

## Introduction

*Staphylococcus aureus* is a major pathogen of human in community and in hospital causing a variety of diseases ranging from mild to life-threatening infections of the skin and soft tissue, bone and joint, surgical wound, indwelling devices, lung and even heart valves (Crossley, 2010).

With the wide-spread dissemination of methicillin-resistant *S. aureus* (MRSA) in hospitals and in communities, treating *S. aureus* associated infections has become increasingly difficult (Blot et al., 2002).

An array of virulence factors, such as Protein A, fibronectin binding protein A/B, α-toxin, β-toxin, δ-toxin, Panton-Valentine leukotoxin (PVL), and phenol-soluble modulins (PSMs), etc., work in concert and contribute to the virulent properties of *S. aureus* (Peacock et al., 2002). The *S. aureus* exotoxins, α-toxin, β-toxin, δ-toxin, PVL, and PSMs lyse leucocytes (Löffler et al., 2010) and α-toxin and PSMs may contribute to the formation of biofilms (Anderson et al., 2012; Schwartz et al., 2012). The surface-associated virulence factors, Protein A, fibronectin binding protein A/B and envelope-associated proteins contribute to the adherence and invasion of *S. aureus* to epithelial cells as well as abscess formation and persistence in host tissues (Cheng et al., 2009). One of the most important virulence factors α-toxin plays a crucial role in pathogenesis and its crucial function in virulence has been demonstrated in animal models with *hla*-defective mutant losing the capabilities to cause diseases (Ohlsen et al., 1997; Kobayashi et al., 2011)—a notion strengthened by the observation that *S. aureus* strains COL, a derivative of *S. aureus* 8325, with defect in accessory gene regulator (*agr*) showed a lower expression of α-toxin and δ-toxin leading to the attenuation of pathogenicity in mouse lethality models (Herbert et al., 2010). Furthermore, some regulators such as *S. aureus* accessory element (SaeRS) (Sacar et al., 2010) and SarA homologs (Cheung and Zhang, 2002; Zielinska et al., 2011) also contribute to the coordinated expression of diverse virulence factors in response to changes in the environment during infection. It is noteworthy to mention that community-acquired MRSA isolates were shown to express more virulence factors than the nosocomial MRSA isolates (Day et al., 2012), underlining the importance of the involvements of virulence factors in bacterial infections and the need for convenient tools for the study of virulence gene expressions.

Thus, the availability of a multiplex promoter reporter platform to monitor in real-time the expressions of a network of selected virulence-associated genes (regulators or virulence factors) will be highly desirable for studying the pathogenesis of the bacteria and for the identification of extrinsic factors regulating its virulence. We report here the construction of a high-throughput compatible *gfp-lux* based multiplex promoter reporter platform to monitor *S. aureus* virulence genes expression simultaneously and in real time, the validation of the platform using extrinsic factors known to interfere with the SarA/agr regulation network, and the application of this platform to successfully identify natural products possessing activities modulating expressions of *S. aureus* virulence genes.

## Materials and methods

### Bacterial strains and plasmids

The bacterial strains and plasmids used or constructed in this study are listed in Tables 1, 2. Luria broth (LB) and LB agar plates were used throughout for growth of *Escherichia coli* and brain heart infusion (BHI) broth and BHI agar plates for *S. aureus*. Chloramphenicol was used at 10 μg/ml and ampicillin was used at 100 μg/ml for plasmid selection. Unless otherwise stated, all cultures were grown aerobically at 37°C with shaking, and growth was monitored at 600 nm with a HITACHI U-2800 (Hitachi, Japan) spectrophotometer.

**Table 1:** **Strains used in this study**.

**Strain**	**Phenotype**	**Comment**	**Source**
**TEST STRAINS**			
RN6390	MSSA, Agr−		A. Cheung
Newman	MSSA, Agr+	SaeS mutant	A. Cheung
COL	MRSA, Agr−		Ho et al., 2008
USA300 FPR 3757	MRSA, Agr+		ATCC ABB1776
AE052	MRSA, Agr+		Ho et al., 2008
**CONTROL STRAIN/CLONE STRAIN**			
RN4220	MSSA		A. Cheung
Top10	*E. coli*		Life Technologies

**Table 2:** **Plasmids used in this study**.

**Plasmid**	**Feature**	**References**
pACL2084	*S. aureus* and *E. coli* shutter plasmid with GFP	Bateman et al., 2001
pAL2	*S. aureus* and *E. coli* shutter plasmid with LuxABCDE	Beard et al., 2002
pGL	pACL2084 backbone with LuxABCDE from pAL2	This study
pGLspa	*spa* promoter amplified from USA300	This study
pGLhla	*hla* promoter amplified from USA300	This study
pGLsaeP1	*saeP*1 promoter amplified from USA300	This study
pGLsaeP3	*saeP*3 promoter amplified from USA300	This study
pGLagrP2	*agrA* promoter amplified from USA300	This study
pGLagrP3	*RNAIII* promoter amplified from USA300	This study
pGLsarA	*sarA* promoter amplified from USA300	This study
pGLsarS	*sarS* promoter amplified from USA300	This study
pGLmap	*map* promoter amplified from USA300	This study
pGLrot	*rot* promoter amplified from USA300	This study
pGLpvl	*lukFS* promoter amplified from USA300	This study
pGLeap	*eap* promoter amplified from USA300	This study
pGLpsm	*psm*-α promoter amplified from USA300	This study
pGLfnbA	*fnbA* promoter amplified from USA300	This study
pGLfnbB	*fnbB* promoter amplified from USA300	This study
pGLsrtA	*srtA* promoter amplified from USA300	This study
pGLclfA	*clfA* promoter amplified from USA300	This study
pGLcap5	Capsular 5 promoter amplified from COL	This study
pGLcap8	Capsular 8 promoter amplified from AE052	This study
pGLami	*ami* promoter amplified from pAL2 plasmid	This study

### DNA manipulations, oligonucleotides, and sequencing

Standard methods for DNA manipulation, preparation, and analysis were employed as described previously (Qazi et al., 2001). Restriction enzymes and T4 DNA ligase were purchased from New England Biolabs (NEB, China) and used in accordance with the manufacturer's instructions. PCR primers (Table 3) were purchased from (Life technologies, Hong Kong). DNA fragments were isolated from electrophoresis agarose (Lonza, Swiss) using gel extraction kit (Qiagen, German). PCR was performed in an ABI thermal cycler 7900 in 50-μL reaction volumes with PrimeSTAR DNA polymerase (Takara, Japan) in accordance with manufacturer's instructions. *E. coli* Top10 cells (Life technologies, Hong Kong) were transformed by heat shock operation. *S. aureus* cells were transformed according to published method (Qazi et al., 2001).

**Table 3:** **Primers used in this study**.

**Gene/promoter^a^**	**Primer for promoter cloning**	**Enzyme site**
*hla*-f	GTTATATGGCTAGCCTCCTGAATTTTTC	*Nhe*I
*hla*-r	ACTTGGAGCTAGCATACGTGTTTTCATTTTCATC	*Nhe*I
*spa*-f	TCCTCGCGCGGCCGCCACTTTATTCTTAAAAA	*Not*I
*spa*-r	GCCTCGCGCTAGCTGTATGTATTTGTAAAGTC	*Nhe*I
*RNAII*-f^b^	TTGCATGCTAGCTTTTACACCACTCTCCTCAC	*Nhe*I
*RNAII*-r	TTGCATGCTAGCCAACTATTTTCCATCACATC	*Nhe*I
*saeP1*-f	GTCGACGCTAGCACTGTTGAAGGTAAAGCTG	*Nhe*I
*saeP1*-r	GTCGACGCTAGCACCTCTGTTCTTACGACC	*Nhe*I
*saeP3*-f	GTCGACGCTAGCTTATTGTGGCAAAAGGTT	*Nhe*I
*saeP3*-r	GTCGACGCTAGCTACCTTGATCTTGTGAAT	*Nhe*I
*sarA*-f	GTCGACGCTAGCATTAACTTTTAGCTTATCATTTTAA	*Nhe*I
*sarA*-r	GTCGACGCTAGCGTTTAAAACCTCCCTATTTGATGC	*Nhe*I
*sarS*-f	GTCGACGCTAGCTGTTTTATCTCCTTGTATATGC	*Nhe*I
*sarS*-r	GTCGACGCGGCCGCCGATATTATTAAAACAAAATG	*Not*I
*lukFS*-PV-f	TTGCATGCTAGCTAATTGTATATGATGAATCTTAG	*Nhe*I
*lukFS*-PV-r	TTGCATGCTAGCAAAAATCATTTCCTTTCTTTA	*Nhe*I
*psm*-f	TTGCATCATGCGGCCGCTAGCTGCATAACCTCCTTATTTC	*Not*I
*psm*-r	TTGCATGCTAGCTAAGATTACCTCCTTTGCTTATGAG	*Nhe*I
*map*-f	TTGCATGCTAGCTTTCAATTATAGTCCGGG	*Nhe*I
*map*-r	CGTGCCTCGCTAGCTTTTATATAATTATAACT	*Nhe*I
*ami*-f	GTGAATTAGCTAGCTAATTTCTATGCGCACCC	*Nhe*I
*ami*-r	ATTCTTGCTAGCTTATAATTACCTCAGGTCG	*Nhe*I
*eap*-f	GTCGACGCTAGCTTTTAATTTCATATAATCTCTCTCC	*Nhe*I
*eap*-r	GTCGACGCTAGCATATACCATATGAATACGCAGCAGG	*Nhe*I
*fnbA*-f	GTCGACGCGGCCGCCTAAATATTAAGTAAACGTG	*Not*I
*fnbA*-r	GTCGACGCTAGCTATAATATCTCCCTTTAAATGC	*Nhe*I
*fnbB*-f	GTCGACGCGGCCGCTTAAACAAAAATTGACGGG	*Not*I
*fnbB*-r	GTCGACGCTAGCTATAATATTCTCCCTTAAATGC	*Nhe*I
*clfA*-f	GTCGACGCGGCCGCTTTCAAGCTAGGATTACATTAGG	*Not*I
*clfA*-r	GTCGACGCTAGCTTTATTCCCTCTTTTTAAAAAGTC	*Nhe*I
*rot*-f	GTCGACGCGGCCGCGGTTGAAAATGTATATCAC	*Not*I
*rot*-r	GTCGACGCTAGCAAAACTACAAGTGTAAATAAAC	*Nhe*I
*srtA*-f	GTCGACGCGGCCGCATAGATTAGTATAGTTAAGGGGGAA	*Not*I
*srtA*-r	GTCGACGCTAGCTGCAATTCCGAGGAAAATATGTAAAGTGT	*Nhe*I
*cap5*/*cap8*-f	TTGCATCATGCGGCCGCGGTCAATCAGTCGGAATT	*Not*I
*cap5*/*cap8*-r	TTGCATGCTAGCCAAGTTTTTTTGTAATA	*Nhe*I
rt-*hla*-f	AAAAAACTGCTAGTTATTAGAACGAAAGG	
rt-*hla*-r	GGCCAGGCTAAACCACTTTTG	
rt-*spa*-f	CAGCAAACCATGCAGATGCTA	
rt-*spa*-r	GCTAATGATAATCCACCAAATACAGTTG	
rt-*RNAIII*-f	GTGATGGAAAATAGTTGATGAGTTGTTT	
rt-*RNAIII*-r	GAATTTGTTCACTGTGTCGATAATCC	
rt-*sae*-f	AAACTTGCTTGATAATGCGCTAAA	
rt-*sae*-r	GTTCTGGTATAATGCCAATACCTTCA	
rt-*sarS*-f	AATACCCTCAAACTGTTAGAGC	
rt-*sarS*-r	TCACTTGAGCTAATAATTGTTCAG	
rt-*sarA*-f	ACATGGCAATTACAAAAATCAATGAT	
rt-*sarA*-r	TCTTTCTCTTTGTTTTCGCTGATG	
rt-*agrA*-f	AAAGTTGCAGCGATGGATTT	
rt-*agrA*-r	ATGGGCAATGAGTCTGTGAG	
rt-*luxA*-f	AGGTCGCATCTCTGAGGAGT	
rt-*luxA*-r	CAATAGCGGCAGTTCCTACA	

a rt refers to RT PCR primers.

b For amplification of RNAIII promoters, reverse the forward and reverse primers.

### Construction of *gfp-lux* dual-reporter plasmids

**Figure 1:**
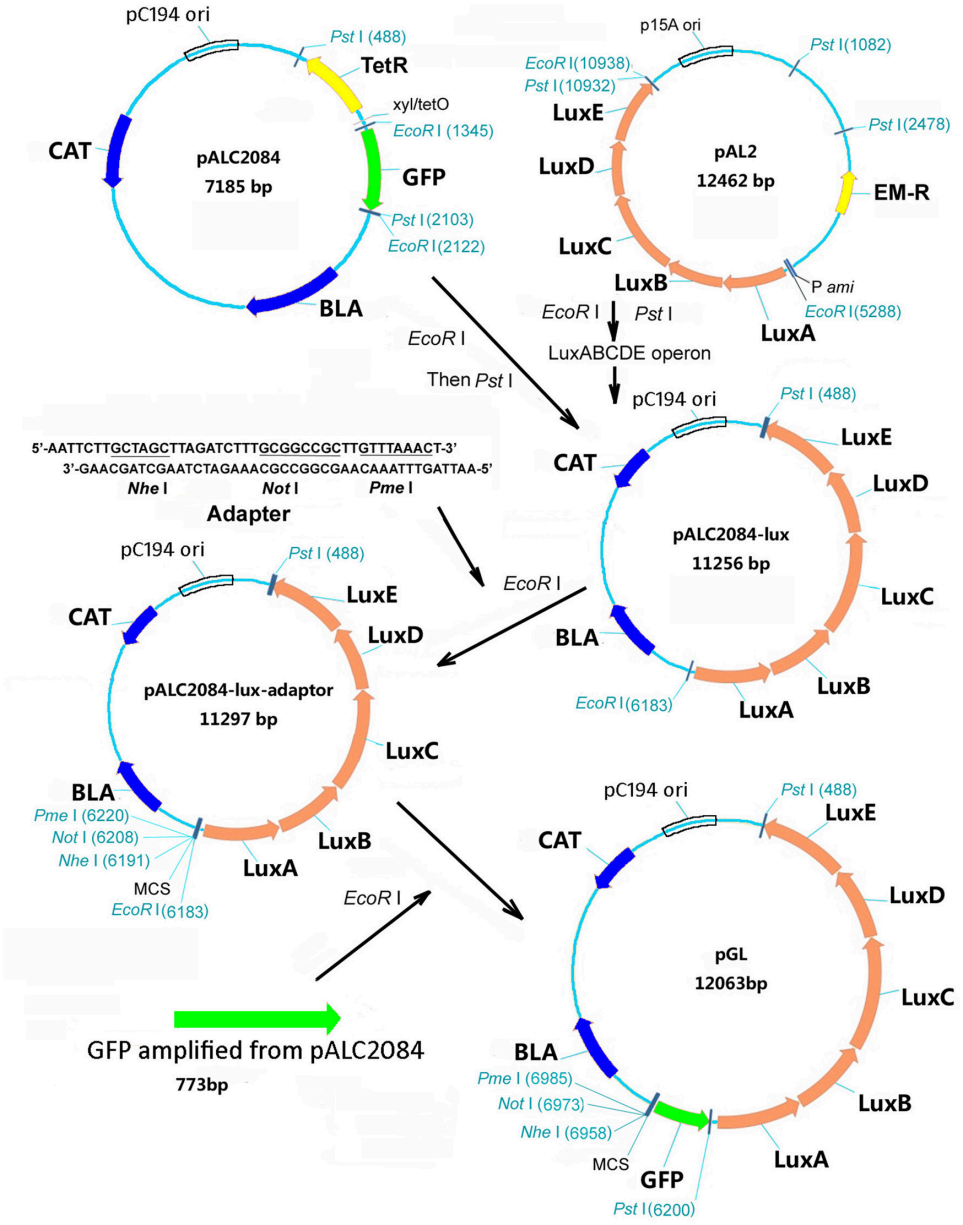
**Schematic diagram showing the construction of plasmid pGL**. Arrows indicate the locations and orientations of open reading frames. The adapter used for adding multiple-cloning sites (MCS) and the restriction enzyme sites were shown. The GFP fragment was obtained from EcoR I digestion of pACL2084. BLA, ampicillin resistance gene; EM-R, erythromycin resistance gene; CAT, chloramphenicol resistance gene.

To construct the *gfp-lux* dual-reporter plasmid, the *luxABCDE* operon was excised from pAL2 as an EcoRI/PstI fragment and inserted into the EcoRI and PstI serial digested plasmid pALC2084 (Bateman et al., 2001), followed by the addition of an adaptor (5′- AATTCTTGCTAGCTTAGATCTTTGCGGCCGCTTGTTTAA ACT-3′) to generate a multiple cloning sites (MCS) with *Nhe*I, *Not*I, and *Nhe*I cleavage sites. The GFP fragment digested from pALC2084 with *EcoR*I was ligated in the upstream of the lux genes without a promoter to generate plasmid pGL (Figure 1). To generate the *promoter*::*gfp-luxABCDE* expression vector, PCR primers (Table 3) were used to amplify the different promoters from *S. aureus* USA300 FPR3757 chromosomal DNA. Each amplicon was restricted with *Nhe*I/*Not*I double-digestion or *Nhe*I single digestion and ligated with pGL digested with corresponding restriction enzyme(s). The direction of ligation product from single digestion was confirmed by PCR. All of the constructions and PCR products were verified by sequencing. The schematic representation of the construction of the plasmids is illustrated in Figure 1.

### qRT-PCR

The preparation of total RNA from *S. aureus* was performed using RNA protection reagent according to the manufacturer's instructions (Qiagen, Germany). Briefly, total RNA was prepared by lysostaphin extraction using 5 × 10^8^ CFU of bacteria at each time point, followed by further purification with an RNeasy kit (Qiagen, Germany) according to the manufacturer's instructions. The quality and quantity of total RNA were confirmed by agarose electrophoresis and UV spectrophotometry, respectively.

Contaminating chromosomal DNA was removed by DNase treatment (Life technologies, Hong Kong). Purified *S. aureus* RNA was reverse transcripted into cDNA by SuperScript® III First-Strand Synthesis SuperMix (Life technologies, Hong Kong) and then subjected to real−time PCR analysis using an ABI 7500 thermocycler (Life technologies, Hong Kong) using Fast SYBR® Green Master Mix (Life technologies, Hong Kong). The relative quantification of *S. aureus* transcripts was determined by the ratio of expression of target transcripts relative to *gyrB* (housekeeping or calibration gene). The sequences of primers for real-time PCR experiments are provided in Table 3.

For the correlation of *lux*A and virulence gene expression, the equation is as follow:

$$\begin{array}{l} \text{Relative expression to gyrB}=\;2^{-\;\left(CT\;target\;gene-CT\;gyrB\right)}=\;2^{-\Delta CT} \end{array}$$

For the compound treatment, the equation is as follow:

$$\begin{array}{l} \text{Normalized relative expression ratio}\\ \;=2^{-\;\left(\Delta CT\;of\;UA\;-\;\Delta CT\;of\;DMSO\right)} \end{array}$$

### Continuously monitoring gene expression in bacterial cultures

For quantification of GFP fluorescence and bioluminescence, overnight bacterial cultures were diluted in appropriate media containing 10 μg/ml chloramphenicol. Samples (100 μL) were transferred into microtiter plate from culture tubes, and fluorescence was measured by using DTX 800/880 multimode plate reader (Beckman). Bacteria with pGL plasmid were included as control to allow correction for background fluorescence.

For bacteria growth curve monitoring, samples (100 μL) with 10^6^ *S. aureus* were separated into aliquots in triplicate into clear-bottom 96-well microtiter plates and incubated at 37°C. The optical density at 620 nm (OD_620_), the fluorescence (GFP), and the bioluminescence were measured every 30min in DTX 800/880 multimode plate reader (Beckman).

### Minimum inhibitory concentration (MICs) tests

MIC was determined by inoculating 5 × 10^4^ *S. aureus* cells in 100 μl BHI media in 96-well plates with a serial dilution of antibiotics. The MIC was defined as the minimum concentration resulting in a cell density less than 0.01 OD at 620 nm (Ohlsen et al., 1998; Ji, 2007), which corresponded to no visible growth, after incubating for 18 h at 37°C.

### Disk diffusion and lux assays

A single colony of bioluminescent *S. aureus* from BHI agar was resuspended in 200 μl of sterile water, diluted to 75 ml 0.7% (w/v) soft agar (375-fold dilution) and overlaid onto BHI plates. Antibiotic disks (Becton Dickinson, Mississauga, ON, Canada; Difco, Detroit, MI, USA) were placed on the overlay and the plates incubated at 37°C. After 20 h, inhibition zones were measured and luminescence was detected with a luminograph LB980 photon camera (Berthold, Oak Ridge, TN, USA) and Xenogen IVIS 100 *in vivo* imaging system (Xenogen, Alameda, CA).

### Screening for repressors of virulence gene expression

The overlay assay was performed with 24.5 cm × 24.5 cm plates (Corning) with 100 paper discs per plate (Mesak et al., 2010). Each paper disc was loaded with 20 μL of different testing samples and incubated at 37°C for 24 h. The luminescence signals were recorded by luminograph LB980 photon camera. We screened 204 crude extracted samples from different sources, such as lichen, tree, moss and Traditional Chinese Medicine (TCM). A total of 9 virulence factors promoters, *srtA, clfA, hla, spa, pvl, psm, fnbB, cap5*, and *cap8* were used for this screen. The screen was repeated twice. Extracts that inhibit the light emission of more than 3 promoters were assigned as hits. Similar screens were also carried out with in-house chemical compounds.

### Adherence assay and invasion assay (Liang and Ji, 2007)

Overnight bacterial culture with or without ampicillin and/or compound treatment were washed 3 times with PBS (pH 7.4) and then diluted to 10^7^ CFU/ml with MEM medium before inoculation (defined as the original bacterial CFU). A549 cells were seeded onto a 24-well tissue culture plate (Greiner) at a concentration of 2 × 10^5^/ml in MEM for counting bacterial adherence and invasion ratio. Briefly, A549 cells were grown overnight at 37°C in 5% CO_2_ to form confluent monolayers. The medium was removed in the following morning and A549 cells were washed twice with 1 ml of PBS, followed by infection with 1 ml of the prepared bacterial inoculum. For invasion assay, after infection of A549 cells at 37°C for 2 h, the supernatants from the wells were collected for total bacterial count (defined as the total bacterial CFU). A549 cells were then washed twice with PBS followed by incubation with MEM containing gentamicin (100 μg/ml; Sigma) and lysostaphin (10 μg/ml; Sigma) for 1 h at 37°C; all wells were then washed 3 times with 1 ml PBS. Subsequently, wells were trypsinized with 150 μl of 0.25% trypsin-EDTA for 5 min, the cells in each well were carefully collected into tubes, and then 400 μl of ice-cold 0.025% Triton X-100 was added to the tubes and put on ice. The numbers of bacterial CFU released from the lysed epithelial cells were determined by plating lysates on BHI agar plates (defined as the invaded bacterial CFU). For adherence assay, after infection of A549 cells at 37°C for 1 h, the medium was removed and A549 cells washed 3 times with 1 ml PBS. Subsequently, a total number of adhered and invaded bacteria released from the lysed epithelial cells was defined as the adhered bacterial CFU.

$$\begin{array}{l} \text{Relative invasion }=\;\frac{\begin{array}{c} \text{Internalized bacteria CFU of sample}/\\ \text{ Total CFU of sample} \end{array}}{\begin{array}{c} \text{Internalized bacteria CFU of control}/\\ \text{ Total CFU of control} \end{array}} \end{array}$$

The bacterial adhesion in each well was determined as the CFU that adhered to and invaded into the cells and is expressed as a percentage of the CFU in the inoculum. The controls were wells pretreated with medium alone (MEM) considered to have 100% adhesion. Adhesion and invasion were then normalized against controls according to the equations.

$$\begin{array}{l} \text{Relative adherence }=\;\frac{\begin{array}{c} \text{Adhered \& Internalized bacteria CFU of}\\ \text{ sample}/\text{Original CFU of sample} \end{array}}{\begin{array}{c} \text{Adhered \& Internalized bacteria CFU of}\\ \text{ control}/\text{Original CFU of control} \end{array}} \end{array}$$

Each experiment was repeated three times, and all of the relative adhesion and invasion values were calculated and statistically analyzed by Student's *t*-test, using SigmaPlot software 11.0. *P* < 0.05 were considered significant (Ji, 2007).

For confocal microscopic analysis of internalized bacteria, A549 cells were seeded onto glass coverslips and incubated at 37°C with 1 ml of the bacterial inoculum for 2 h. After the removal of external bacteria, image capture was done using a Zeiss LSM 700 Inverted Confocal Microscope (Carl Zeiss, Jena, Germany). In order to perform quantitative analysis, a minimum of 5 fields per slide were examined.

## Results

### Construction of plasmid pGL and ligation of different promoters into pGL

Figure 1 is a schematic illustration of the construction of plasmid pGL confirmed by restriction enzyme digestion and sequencing. In this construct, *gfp* and *lux* genes were cloned in an operon and co-expressed from the promoters of interest. GFP amplified from pALC2084 was used here for investigating the accumulation of gene products expressed. The *lux* gene cluster from pAL2 was used for monitoring the real-time gene expression. Transformation frequency of pGL in *S. aureus* RN4220 and *S. aureus* USA300 was 1.3 × 10^4^/μg and 8.1 × 10^3^/μg respectively. Antibiotic resistance markers used were ampicillin in *E. coli* and chloramphenicol in *S. aureus*. Promoters of 18 regulators and virulence factors (Table 4), *map* promoter and *ami* promoter from pAL2 were amplified and ligated into plasmid pGL. All constructs were confirmed by DNA sequencing.

**Table 4:** **Promoters used for the construction of 20 *gfp*-*luxABCDE* dual-reporter plasmids**.

**No**.	**Gene promoters**	**Gene product**	**Role in virulence**
**REGULATORS**			
1	*agr*	Accessory gene regulator	Quorum sensing
2	*rot*	Repressor of toxin	Repress toxins
3	*sarS*	Staphylococcal accessory regulator	Regulate *spa*
4	*saeP1*	*S. aureus* exoprotein expression regulator SaePQRS	Regulation of exotoxins
5	*sae P3*	*S. aureus* exoprotein expression SaeRS	Regulation of exotoxins
6	*sarA*	Staphylococcal accessory regulator	Regulation *agr* and extracellar and surface-associated virulence factors
7	*RNAIII /hld*	Regulator/δ-toxin	Sensing/cell lysis
**EXOTOXINS**			
8	*hla*	α-toxin	Cell lysis
9	*pvl*	Panton-Valentine leukotoxin	Cell lysis
10	*psm*	Phenol-soluble modulins	Cell lysis
**SURFACE TOXINS**			
11	*spa*	Protein A	Inhibits opsonophagocytosis
12	*eap*	Extracellular adhesion protein	Wound healing
13	*fnbA*	Fibronectin binding protein A	Adhesion: fibrinogen
14	*fnbB*	Fibronectin binding protein B	Adhesion: fibrinogen
15	*clfA*	Clumping factors A	Adhesion: fibrinogen, nasal colonization, Evasion of phagocytosis
16	*cap5*(COL)	Type 5 capsular polysaccharide	Inhibits opsonophagocytosis
17	*cap8*(AE052)	Type 8 capsular polysaccharide	Inhibits opsonophagocytosis
**ENZYME**			
18	*srtA*	Sortase A	Anchor cell wall surface protein
**CONTROLS**			
19	*map*	Methionine aminopeptidase	–
20	*ami*	Aminopterin resistance operon	

The plasmids were successfully constructed and introduced into *S. aureus* strains by electroporation. The transformants were confirmed by plasmid isolation and fluorescence and bioluminescence readings in liquid culture.

### Monitoring gene expression of *S. aureus* using luminescence and fluorescence

**Figure 2:**
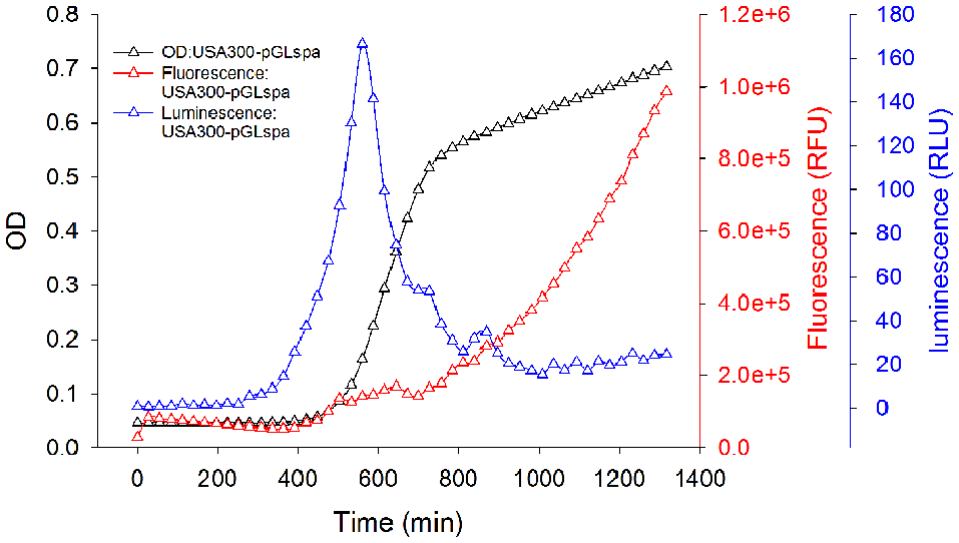
**The correlation of bacterial growth and gene expression monitored by luminescence and fluorescence**. Bacteria harboring plasmid pGLspa were inoculated in a black 96-well microtiter plate with clear bottom (Greiner bio-one, German) and OD, luminescence, and fluorescence were monitored for every 30 min using a DTX880 multimode plate reader (Beckman Coulter). Blue triangle, luminescence signal of bacteria with plasmid pGLspa; Black triangle, OD reading of bacteria with plasmid pGLspa; Red triangle, fluorescence signal of bacteria with plasmid pGLspa.

When bioluminescence was monitored in *S. aureus* USA300-pGLspa grown in liquid culture, gene expression driven by *spa* promoter peaked during exponential phase and diminished when reaching post-exponential phase (Figure 2), similar to what was reported earlier (Gao and Stewart, 2004). Consistent with the study reported by Qazi et al. (2001), GFP fluorescence was detected later during the growth of the bacteria, and the fluorescence signal increased for a longer period of time (Figure 2). This observation may due to a lag period needed for GFP folding and the well-known stability of GFP (>24 h). Our data were in accordance with the results from Qazi (Qazi et al., 2001) that Lu*x* may serve as a real-time reporter of promoters while GFP may be used for the observation of accumulation effects of gene expression. The combination of both reporters in a dual-reporter system will be appropriate for carrying out high-throughput screening for unknown repressors or inducers of promoters.

### Correlation of lux signal with *S. aureus* gene expression and *luxA* expression

**Figure 3:**
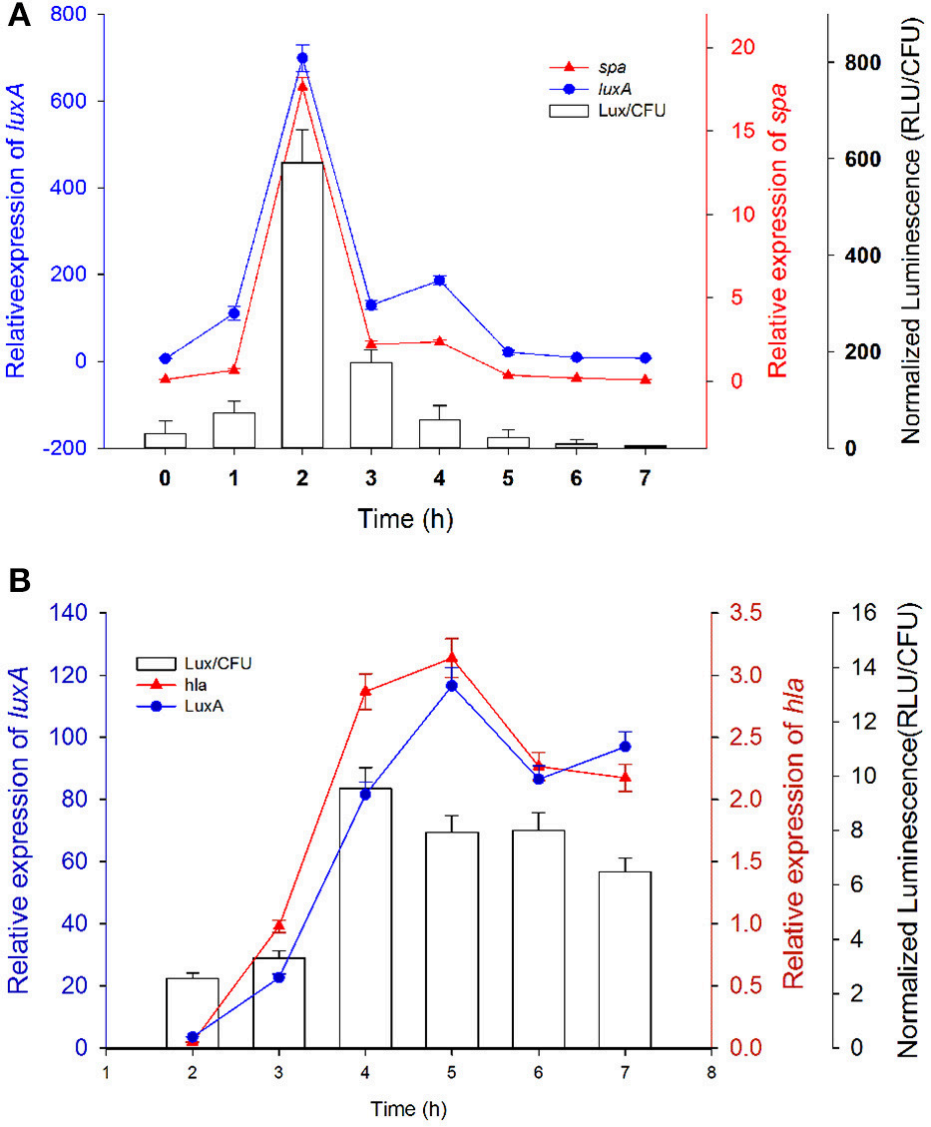
**Correlations between the gene expression monitored by real-time PCR of *spa* and *luxA* driven by the *spa* promoter and the expression of the same promoter monitored by luminescence signal**. Bacteria containing plasmid pGL-*spa* **(A)** or pGL-*hla* **(B)** were cultured with shaking at 37°C and luminescence signals were monitored every 1 h. RNA was extracted at corresponding time point for quantifying the gene expression level by real-time PCR. Bar graph, normalized luminescence signal; Red solid triangle, relative *spa/hla* expression; Blue solid cycle, relative *luxA* expression. Experiments were carried out in triplicate and repeated twice. The mean value is shown with s.d.

Taking protein A as an example, expression driven by the *spa* promoter showed a similar phase-dependent effect as reported (Gao and Stewart, 2004), the correlation of luminescence signal, *LuxA* gene expression, and *spa* expression was confirmed in our system. Using the house-keeping *gyrB* gene as a standard, the relative ratio of *luxA* and *spa* expression was calculated, plotted in Figure 3 and correlated with luminescence signal normalized by CFU. As shown in Figure 3, the luminescence signal fully represented the expression pattern of *luxA* and *spa* gene, which were all under the control of *spa* promoter. The correlation among relative expressions of *lux*A, virulence gene, and normalized luminescence indicated that the luminescence signal can represent the endogenous virulence gene expression.

### The effects of extrinsic factors on the expression of selected *S. aureus* promoters

**Figure 4:**
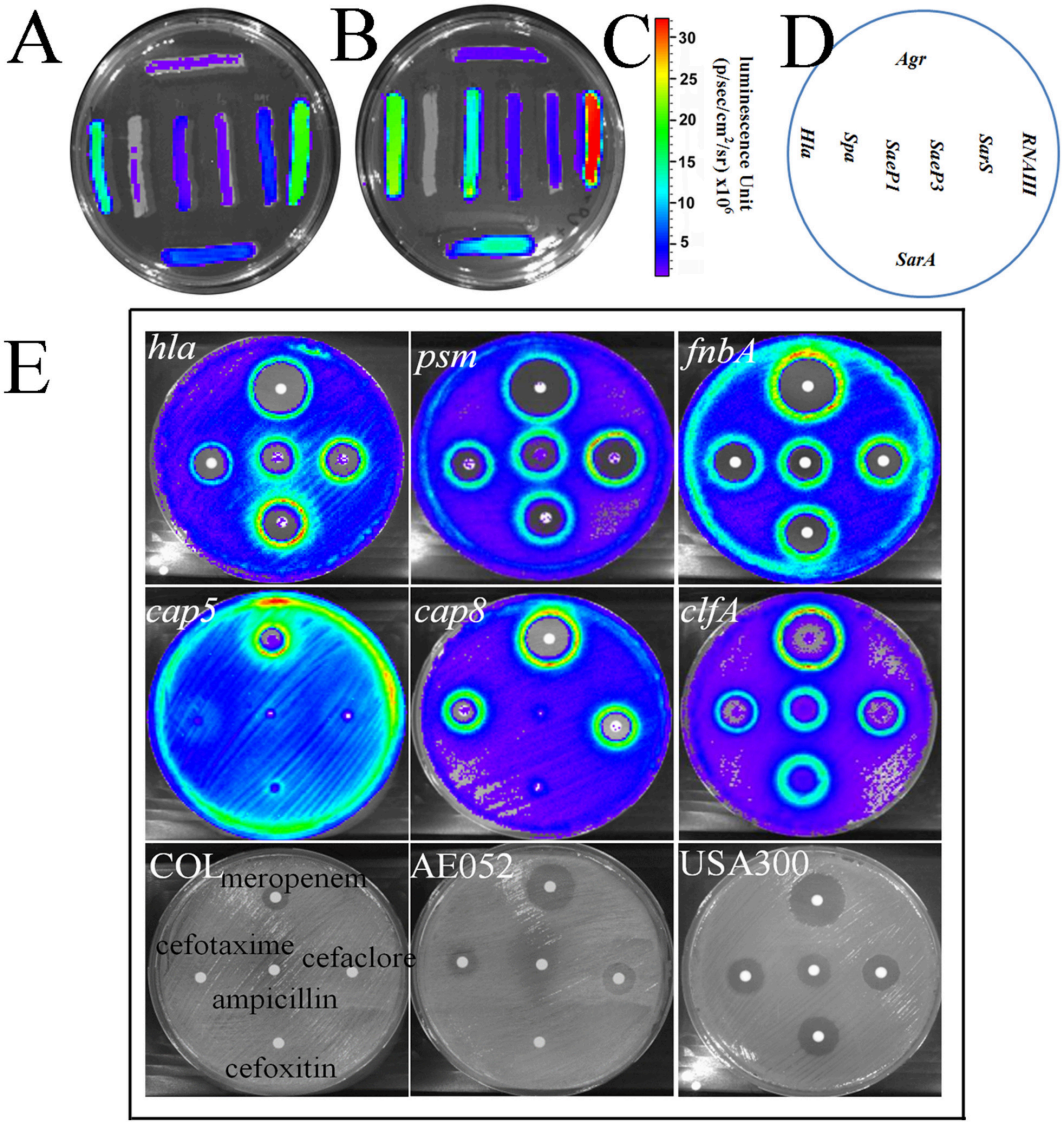
**CO_2_ affected the expression of different gene promoters**. Bacteria carrying reporter systems with different promoters were streaked onto BHI-agar plates and incubated at 37°C with or without 5% CO_2_. The signals were monitored by an IVIS 100 *in vivo* imaging system (Xenogen, Hopkinton, MA). **(A)** The plate incubated without 5%CO_2_. **(B)** The plate incubated with 5% CO_2._**(C)** Color bar denoting the intensity of luminescence signals. **(D)** Distribution of streaks of bacteria harboring different promoters on the agar plate. **(E)** Different induction effect of β-lactam antibiotics on various virulence factors' promoters.

After confirming the relationship between luminescence and gene expression, a well-studied environmental factor CO_2_ (Ohlsen et al., 1997) was used to analyze the CO_2_ effects on the expression of 8 genes (*agr, sarA, hla, spa, saeP1, saeP3, sarS*, and *RNAIII*) closely related to *S. aureus* virulence (Schmidt et al., 2003). Results showed that CO_2_ affected the expression level of protein A by repressing the activity of the *spa* promoter and inducing the expression of *hla* gene (Figure 4A); as reported by Ohlsen et al. (1997). As *sarA* and *agr* regulate *hla* and *spa* (Schmidt et al., 2003), in this pathway, up-regulated *sarA* may repress *spa* expression and induce *hla* expression directly and indirectly through inducing *agr* (*RNAIII*) to repress *sarT* expression, which may further induce *hla* expression and repress *spa* expression (Figures 4A–D). Furthermore, consistent with the report of Herbert et al. (1997), activities of type 5 promoter was reduced in the presence of CO_2_ (Figure S1). The platform may be used for the illustration of the interplay between regulators and virulence factors.

It has been shown that sub-inhibitory concentrations of β-lactam antibiotics strongly induce *hla* expression (Ohlsen et al., 1998). Applying this platform, 5 different β-lactam antibiotics were tested on different virulence factors' promoters (Figure 4E). Meropenem, cefotaxime, cefaclor and cefoxitin target penicillin-binding protein 1 (PBP1), PBP2, PBP3, and PBP4 respectively and have shown induction effect on *hla, psm, clfA, fnbA, cap*5, and *cap*8 at subinhibitory concentrations. The induction effect was also monitored on ampicillin. We observed different induction level of these antibiotics on cap5 promoter and different induction level of one antibiotic on different promoters' activities. The induction of *clfA* and *fnbA* may lead to induced adherence and internalization. The platform may be used for the investigation of modulating effects of extrinsic factors on virulence gene expression.

### Ampicillin induces adherence and invasion of *S. aureus*

**Figure 5:**
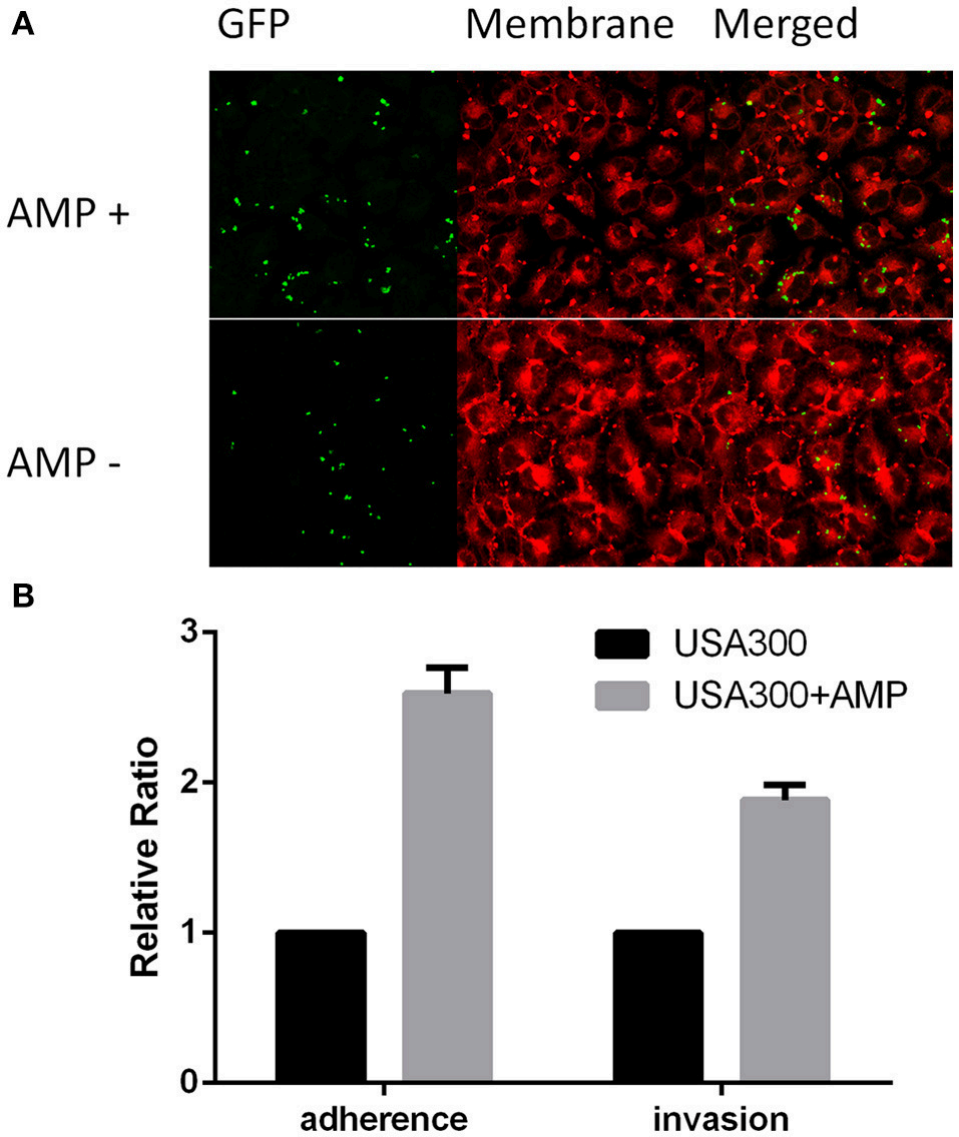
**Effects of ampicillin on the adherence and internalization of *S. aureus* USA300 by A549 human lung epithelial cells**. Relative invasion and relative adherence assays were performed in triplicate and repeated twice. **(A)** Ampicillin enhances the adherence of USA300 toward A540 cells. USA300-pGLami was cultured overnight in the absence of ampicillin (AMP−) or with 1/16 MIC concentration of ampicillin (AMP+) followed by co-culturing with A549 cells for 1 h. USA300 expressing GFP are shown in green. **(B)** Sub-inhibitory concentration of ampicillin induced adherence ratio and invasion ratio of USA300 in A549 cells. Experiments were carried out in triplicate and repeated twice. The mean value is shown with s.d.

As ampicillin was shown to induce nearly all of the tested virulence-related promoters (Figures 4B, **7B** and Table 5), we tested if sub-inhibitory concentrations of ampicillin increase invasiveness of *S. aureus* in epithelial cells. We prepared USA300 overnight culture with or without ampicillin treatment to infect A549 cells (human lung epithelial cells) so as to evaluate the cell adherence and cellular invasion of the bacteria. To illustrate the enhanced intracellular localization of the bacteria by ampicillin in the invasion assay, GFP fluorescence from the engineered bacteria was used to trace the localization of the bacteria. Images taken from confocal fluorescence microscope indicated that the bacteria were internalized into the A549 cells and ampicillin treatment apparently increased the number of bacteria internalized (Figure 5A). After calculating the ratio of bacteria adherence and internalization of *S. aureus* cells (Ji, 2007), the bacteria adherence ratio and bacterial invasion ratio of ampicillin-treated bacteria were considerably higher than that without the ampicillin treatment (Figure 5B). The induction effect on adherence factors at subinhibitory concentrations of ampicillin has led to induced adherence and invasion of the bacteria.

**Table 5:** **Effects of three natural extracts on selected virulence-related promoters**.

**Promoters**	**15**	**AE62D**	**AE63**	**AMP**
*spa*	−	−	+++	++
*pvl*	−	−	−	++
*hla*	−	−	−	++
*psm*	−	−	−	+
*fnbB*	−	−	+	+
*cap8*	+	−	++	+
*cap5*	+	−	+	+
*sarS*	−	−−	+	+
*saeP1*	−	−	−	+

+, induction effect; −, repression effect. 15, an extract from Abies grandis (grand fir); AE62D, an extract from Sphaerophorus globosus (coral lichen); AE63, an extract from Usnea filipendula (beard lichen); AMP, ampicillin.

### Screening for virulence repressors

**Figure 6:**
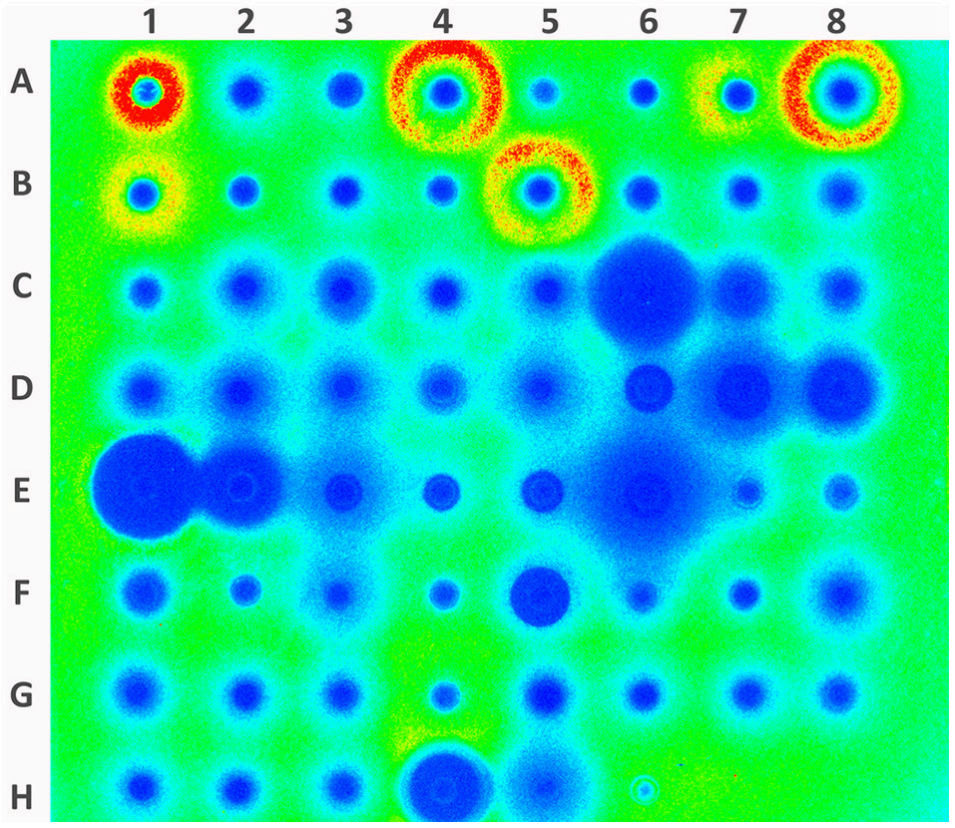
**Screening promoter-repressing compounds with hla promoter from 208 natural products**. The paper disc with natural product samples distributed from A1 to H3. Paper discs with ampicillin (10 mg/ml) and Ethyl acetate were loaded on H4 and H6, respectively. The blue color indicates repression of promoter activity and red color indicates induction of promoter.

**Figure 7:**
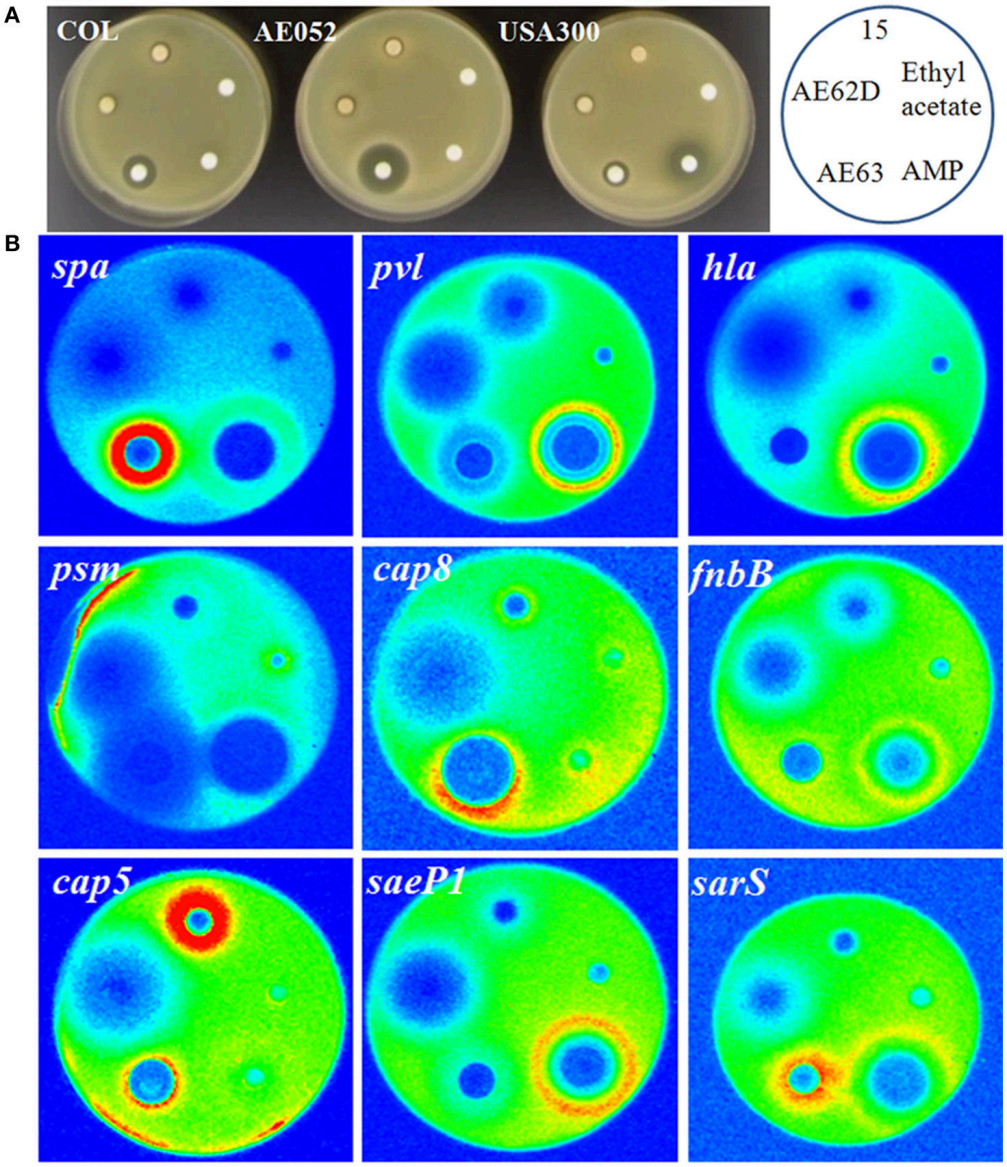
**Natural extracts affecting the expression of selected virulence-related genes in different *S. aureus* strains. (A)** Inhibition zones of selected extracts on three different *S. aureus* strains, USA300, COL, and AE052. Samples 15, AE62, and AE63 are natural products exerting varying degree of repression of virulence gene in USA300. **(B)** The response of promoters of 9 virulence-related genes to three selected natural products. Ampicillin and ethyl acetate/DMSO were taken as positive and negative controls respectively.

The multiplex promoter reporter platform was used directly for screening potential virulence repressors from a natural products library. We used COL with plasmid pGLcap5, strain AE052 with plasmid pGLcap8 and strain USA300 with other plasmids to screen compound libraries. For the crude extracted samples from different sources, such as lichens, tree, mosses and TCM, used in the screening (Figure 6), 20 samples were found to reduce luminescence signals of more than 3 (out of the 7) different promoters. Five hits out of 208 samples were showing the suppressing effect on more than 4 promoters and 13 hits showing the suppressing effect on all the 9 promoters. We then selected 3 samples with different repression profiles showing their repression effects on 7 selected virulence-related promoters (*hla, spa, pvl, psm, fnbB, cap5*, and *cap8*, Figures 7A,B). The data showed that sample 15 extracted from *Abies grandis* (grand fir) reduced the activity of 5 promoters except *cap5* and *cap8*; sample AE62D extracted from *Sphaerophorus globosus* (coral lichen) reduced expression of all 7 promoters tested and sample AE63 extracted from *Usnea filipendula* (beard lichen) reduced exotoxins, for example, *hla, pvl*, and *psm* but induced luminescence signal of cell surface-associated virulence factors like *spa, fnbB, cap5*, and *cap8* (Table 5).

### Usnic acid suppresses virulence factors expression

**Figure 8:**
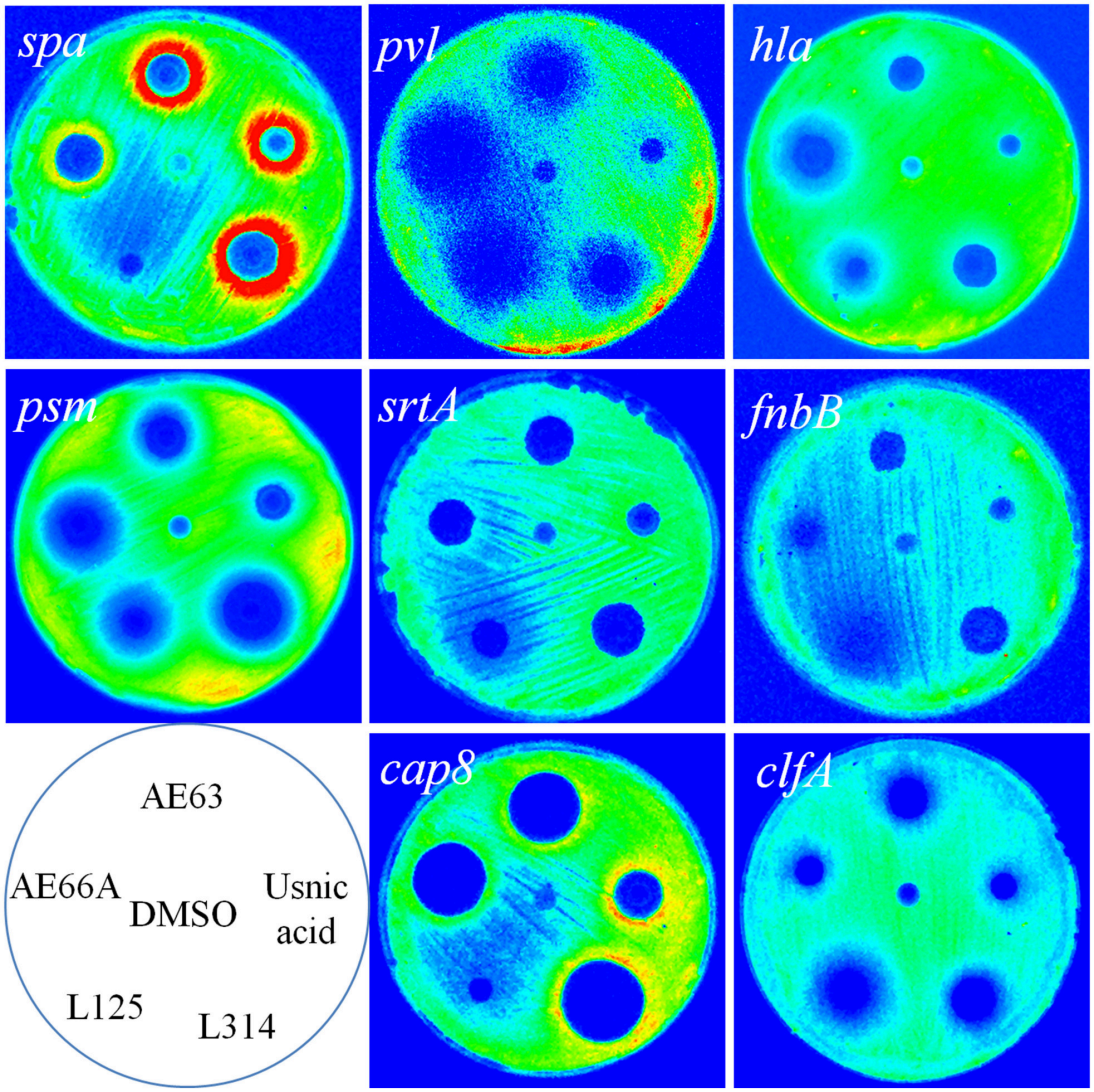
**Correlation between natural extracts and usnic acid on different virulence factors promoters**. The response of promoters of 8 virulence-related genes to four selected natural products and usnic acid. DMSO was taken as negative controls.

Among the 5 hits showing inhibition effect on more than 4 promoters, 3 of them were extracts from Usnea species, namely *Usnea subfloridana, Usnea filipendula, Usnea rigida*, and the other two were from *Sciadopitys verticillata* and *Cryptomeria japonica*. The effective patterns of promoters activities were shown in Table 6. As 4 of them were from usnea, and usnic acid is uniquely found in lichens and is especially abundant in genera such as *Alectoria, Cladonia, Usnea, Lecanora, Ramalina*, and *Evernia*, usnic acid may be the common effective component in these samples. The effect of Usnic acid was also monitored compared with crude extracts from lichens. As shown in Figure 8, usnic acid nearly replicated the relative activity of difference extract. They also showed repression effects on *psm, hla, pvl*, and *clfA*, while induction effects on *spa* and *cap*8. These results indicated that the main effective component in these lichens may be usnic acid.

**Table 6:** **Effects of Usnic acid and four natural extracts on selected virulence-related promoters**.

**Genus**	**species**	**Extract**	***spa***	***srtA***	***psm***	***hla***	***cap8***	***pvl***	***clfA***	***fnbB***
*Usnea*	*subfloridana*	L125	−	−−	−−	−−	−	−−−	−−	−
*Usnea*	*subfloridana*	L314	+++	+	−−	−−	++	−−	−−	/
*Usnea*	*filipendula*	AE63	+++	−	−−	−−	++	−−−	−−	+
*Usnea*	*rigida*	AE66A	++	−	−−	−−	+	−−−	−−	−
Compound		Usnic acid	+++	+	−	−	+	−−	−−	/
Control		DMSO	/	/	/	/	/	/	/	/

+, induction effect; −, repression effect; /, no effect.

### Usnic acid mainly suppresses *psm* expression in CA-MRSA

**Figure 9:**
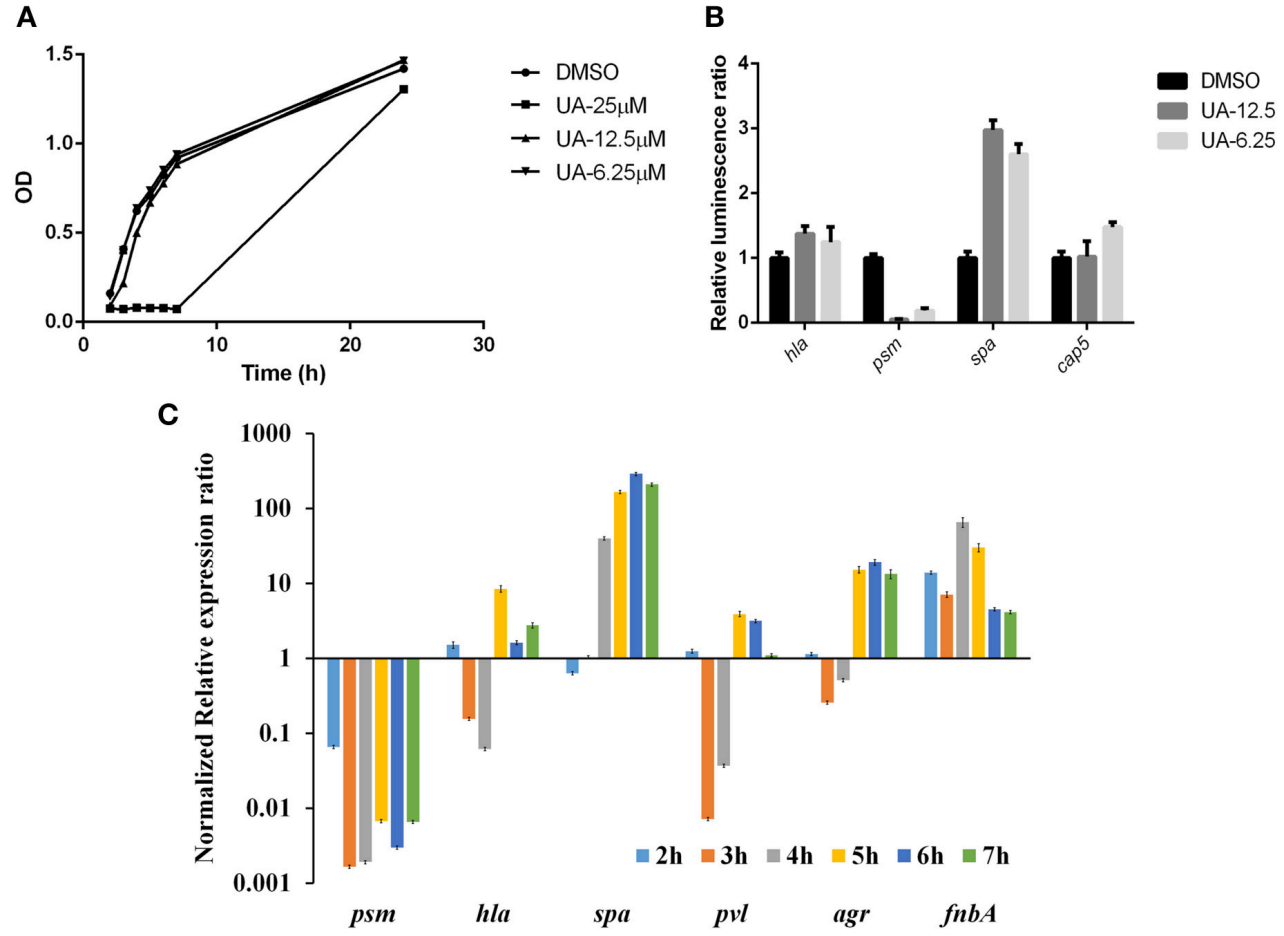
**Usnic acid effect on *S. aureus* growth, luminescence signal of selected virulence factors and different genes expression by RT-PCR. (A)** Growth curves of *S. aureus* after exposure to various concentrations of usnic acid. **(B)** Four selected promoters' activities affected by 12.5 and 6.25 μM usnic acid. **(C)** RT-PCR analysis of 12.5 μM usnic acid on various virulence genes expression at different time points. Data were normalized to DMSO.

We also monitored the growth of USA300 with different concentration of usnic acid, and 12.5 μM usnic acid did not affect bacterial growth while the MIC was around 25 μM (Figure 9A). By analyzing the promoter activity monitored by luminescence signal, we found that usnic acid mainly reduced expression of *psm* (Figure 9B). This was confirmed by real time-PCR results in Figure 9C. In all the time points, the expression of *psm* was reduced for more than 100-fold, while *spa* and *fnbA* were induced for more than 100-fold. In some of them, *hla* and *pvl* were repressed, indicating a regulating network among these virulence factors.

## Discussion

Compared with other widely used reporter systems such as *lacZ* (β-galactosidase), *xylE* (catechol 2,3-dioxygenase), and *blaZ* (β-lactamase), the *lux* (luciferase) and GFP (green fluorescent protein) are more amendable for real-time *in vitro* and *in vivo* experiments. As we have successfully constructed a *gfp-lux* dual-reporter system driven by various virulence gene promoters. This dual-reporter system can be used easily to monitor the virulence gene expression in real-time, the accumulation of gene products, and the growth of the bacteria. We used the expressions of virulence genes and regulators in SarA/agr regulation web to validate our system by studying the effect of extrinsic factors involved in the regulation of virulence gene expressions. According to the SarA/agr regulation network proposed by Cheung (Cheung and Zhang, 2002), *agr* (RNAIII) is involved in the regulation of *hla* and *spa*. Our data show that CO_2_ can induce the expression of *hla* promoter and at the same time repress that of spa, reconciling well with the proposed regulation of the pathway (Cheung et al., 2001) and validate the usefulness of our multiplex promoter reporter platform in determining the expression levels of targeted gene promoters.

Due to the differential diffusion rate and distribution of antibiotics in tissues and organs, it is very likely that in patients antibiotics may not reach the required concentrations to eliminate MRSA, and some population of the bacteria would be subjected to sub-inhibitory antibiotics effects (Dancer, 2008). It is evident from this study and others (Ohlsen et al., 1998; Kuroda et al., 2007; Stevens et al., 2007) that sub-inhibitory concentrations of β-lactams stimulate the expression of many *S. aureus* virulence genes. In this work, after treating various strains of *S. aureus* with ampicillin, the expression of *spa, fnbA, fnbB, cap5, cap8*, and *srtA* were induced. When ampicillin-treated or untreated MRSA were used to infect epithelial cells, *S. aureus* adherence and invasion were enhanced in bacteria treated with sub-lethal dosages of ampicillin, suggesting the possibility that the severity of MRSA infections may increase if patients are treated with sub-inhibitory concentrations of β-lactam antibiotics.

As the virulence of *S. aureus* arises from a combination of several surface-associated virulence factors, exotoxins, enterotoxins and superantigens (Crossley, 2010), and virulence expression is a highly regulated and concerted process influenced by various known regulators, such as *agr, sar*, and *sae*, and also unknown regulators yet to be discovered, knocking down of one virulence-related pathway might have consequences affecting the expression of other virulence factors. For example, a mutation in *agr* eliminates α-toxin expression but caused a burst of the expression protein A (Gao and Stewart, 2004). It has been reported that thymol (Qiu et al., 2010b), eugenol (Qiu et al., 2010a), and perilla oil (Qiu et al., 2011) can significantly reduce the *S. aureus* virulence through reducing the expression of *hla* and some other virulence factors. With the multiplex promoter reporter system well-tested with known factors perturbing the expression of relevant virulence genes and published regulatory circuits, rapid identification of novel compounds or factors inhibiting various virulence genes may be achieved. We have carried out screening using this platform and have identified natural products that can repress the luminescence signal of several promoters simultaneously, implying the feasibility of suppressing multiple virulence factors simultaneously using a small-molecule compound approach. Usnic acid was identified to suppress the expression of multiple *S. aureus* virulence genes at sub-inhibitory concentration, especially for *psm* gene. Psm was reported to contribute to the biofilm formation of *S. aureus* (Schwartz et al., 2012) and this suppression effect of usnic acid on *psm* may explain the inhibition effect of usnic acid on biofilm formation of *S. aureus* (Francolini et al., 2004). Usnic acid has been proposed as an antibacterial agent targeting RNA and DNA synthesis (Maciag-Dorszynska et al., 2014), the reduced virulence factor expression at subinhibitory concentrations may enlarge the window of clinical application. It is plausible that usnic acid may be used single or combined with antibiotics to treat bacterial infections.

With the availability of this multiplex promoter reporter platform to monitor *S. aureus* virulence genes expression, we have demonstrated the possibility of identifying compounds that suppress the expression of a consortium of virulence factors in *S. aureus*.

## Author contributions

PG constructed and validated the platform. PG and YW conducted the adherence and invasion experiment. PG and IV did the screening of natural crude products. PH, RK, and PG designed the experiment. PG and RK draft the manuscript. PH and JD contributed to the draft and finalization.

### Conflict of interest statement

The authors declare that the research was conducted in the absence of any commercial or financial relationships that could be construed as a potential conflict of interest.
