# Supplementary material for: Construction of a Multiplex Promoter Reporter Platform to Monitor Staphylococcus aureus Virulence Gene Expression and the Identification of Usnic Acid as a Potent Suppressor of psm Gene Expression
Source: Front Microbiol. 2016 Aug 30;7:1344. doi: 10.3389/fmicb.2016.01344 (PMC5004274; doi:10.3389/fmicb.2016.01344)
Supplement: Supplementary file 1 [file Image1.PDF]

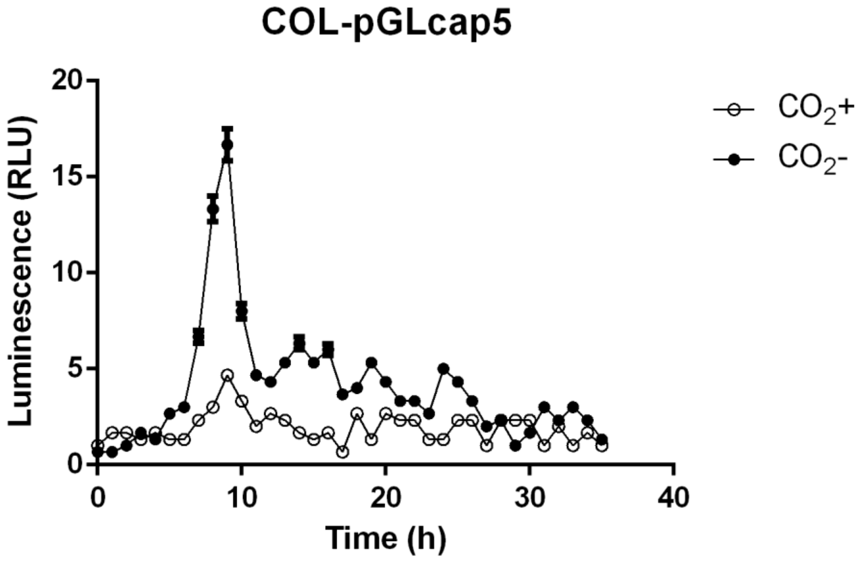

Comparison of the effects of CO_2 on the activity of *cap*5 promoter. COL-pGL-*cap5* (CFUs=2.0E6)were inoculated in a black 96-well microtiterplate withaclear bottom (Greiner Bio-One), incubated at 37°C with or without CO_2,and the OD, luminescence and fluorescence were monitored every 30 min using a DTX880 multimode plate reader (Beckman Coulter). Experiments were conducted in triplicate and repeated twice.
